# The Use of Census Migration Data to Approximate Human Movement Patterns across Temporal Scales

**DOI:** 10.1371/journal.pone.0052971

**Published:** 2013-01-09

**Authors:** Amy Wesolowski, Caroline O. Buckee, Deepa K. Pindolia, Nathan Eagle, David L. Smith, Andres J. Garcia, Andrew J. Tatem

**Affiliations:** 1 Department of Engineering and Public Policy, Carnegie Mellon University, Pittsburgh, Pennsylvania, United States of America; 2 Department of Epidemiology, Harvard School of Public Health, Boston, Massachusetts, United States of America; 3 Center for Communicable Disease Dynamics, Harvard School of Public Health, Boston, Massachusetts, United States of America; 4 Emerging Pathogens Institute, University of Florida, Gainesville, Florida, United States of America; 5 Department of Geography, University of Florida, Gainesville, Florida, United States of America; 6 Malaria Public Health and Epidemiology Group, Centre of Geographic Medicine, KEMRI-Wellcome Trust-University of Oxford Collaborative Programme, Nairobi, Kenya; 7 Department of Computer Science, Northeastern University, Boston, Massachusetts, United States of America; 8 Department of Epidemiology, Johns Hopkins Bloomberg School of Public Health, Baltimore, Maryland, United States of America; 9 Fogarty International Center, National Institutes of Health, Bethesda, Maryland, United States of America; New York State Museum, United States of America

## Abstract

Human movement plays a key role in economies and development, the delivery of services, and the spread of infectious diseases. However, it remains poorly quantified partly because reliable data are often lacking, particularly for low-income countries. The most widely available are migration data from human population censuses, which provide valuable information on relatively long timescale relocations across countries, but do not capture the shorter-scale patterns, trips less than a year, that make up the bulk of human movement. Census-derived migration data may provide valuable proxies for shorter-term movements however, as substantial migration between regions can be indicative of well connected places exhibiting high levels of movement at finer time scales, but this has never been examined in detail. Here, an extensive mobile phone usage data set for Kenya was processed to extract movements between counties in 2009 on weekly, monthly, and annual time scales and compared to data on change in residence from the national census conducted during the same time period. We find that the relative ordering across Kenyan counties for incoming, outgoing and between-county movements shows strong correlations. Moreover, the distributions of trip durations from both sources of data are similar, and a spatial interaction model fit to the data reveals the relationships of different parameters over a range of movement time scales. Significant relationships between census migration data and fine temporal scale movement patterns exist, and results suggest that census data can be used to approximate certain features of movement patterns across multiple temporal scales, extending the utility of census-derived migration data.

## Introduction

Human movement affects important processes in the fields of public health, economics, and ecology. For example, the progression of epidemics and maintenance of endemic diseases are strongly linked to human movement patterns [1]–[7]. Economic development can be driven by access to markets and efficient transportation to increase workforce mobility and the flow of goods [8]–[9]. Moreover, increasing human mobility has lead to the dispersal of exotic species around the world, causing significant economic damage in the case of pest species [10]–[11]. Planning, mitigation, and development policies can be better informed through the incorporation of data on human movement.

The measurement of human movement patterns is notoriously difficult, however, and reliable datasets are few and far between, especially in low-income regions of the world. Data on movement are often collected for specific purposes that restrict generalizability. For instance, traffic data are often collected for specific development purposes such as the building of a new road [8], while commuting to work surveys range in scope and sample size [5]–[6], [12]–[14], but are mostly limited to high-income countries and those making specific workplace trips. Travel history questions from household surveys provide highly detailed data about an individual’s movement patterns, but rarely sample more than a hundred individuals from a restricted group, and often suffer from recall bias [15]–[17].

The most widely used form of human movement data across large areas is generated by national population and housing censuses, available for almost all countries worldwide. A standard census question asks respondents about their place of residence one year previously. Responses to this question are often used to derive estimates of rates of migration across or between countries [18]–[20]. It remains unclear to what extent these migration data represent and relate to the more frequent movements over shorter time periods, for instance trips lasting a week or a few months, that are of importance to factors such as disease spread and economic development. The long term movements captured in census data may well provide valuable proxies for shorter term movements, however, and strong migration links between regions might be indicative of well-connected locations that also exhibit high levels of movement at shorter time scales because people from a given population may be more likely to migrate to places that are well visited by themselves and others from the same population. Although this assumption is often held, it has never been validated.

Mobile phone usage data has recently been shown to be a valuable source for information on short-term frequent movements. The call data records (CDRs) provide the location of the user at the time that they make a call or text, proving a high temporal and spatial resolution picture of large samples of individual movements over time periods of a year or more, and have been shown to be valuable representations of human movement patterns over temporal and spatial scales unachievable with other types of data [7], [21]–[27]. Such datasets are not widely available, however, are difficult to obtain, and are highly sensitive, making them difficult to share and analyze for most countries and time periods. The data can be used to examine the relationships between the short-term movements captured by the mobile phones, and the longer term movements captured by the freely and widely available census data, and potentially extend the utility of census migration data, enabling the development of a better understanding of multi-scale human movement patterns across large areas.

Here we use a comprehensive mobile phone usage dataset for Kenya describing the movement of almost 15 million users, derived from their 12 billion communications over the course of a year, to examine the extent to which census migration data from a similar time period represents movements across a range of temporal scales in absolute and relative terms. Moreover, we explore the fit of gravity-type spatial interaction models to the datasets to examine the potential of using such models for quantifying movement patterns based on geographically referenced demographic data.

## Materials and Methods

### Data

#### Mobile phone usage data

Anonymized mobile phone call record data aggregated to routing tower level for Kenya were provided by the incumbent mobile phone provider and included the timings of calls and SMS communications from 14,816,512 subscribers from June 2008 - June 2009 (with February 2009 missing from the data set). In the interest of protecting privacy, limited access to the anonymized data was made available to a select set of researchers. Following the precedent of previous similar studies [7], [21]–[27], the data were provided in an anonymized form, with subscribers represented as unique hashed IDs, and were processed in a similar manner to those previous studies. In total over twelve billion mobile phone communications were recorded including the location of one of 11,920 routing towers. The operator who provided the call data records had approximately 92% market share at the time of data acquisition. All subscriber data was aggregated to the county level scale to further preserve anonymity.

#### Census data

In 2009, Kenya conducted a national population and housing census. From the census results, data on the number of residents who changed residence between all 48 counties during the previous year were obtained.

#### Quantifying movement

The mobile phone data is presented as call data records (CDRs). Each entry in a CDR contains an anonymized caller ID, anonymized receiver ID, date, duration, and tower routing number for both the caller and receiver. From the CDRs the geographic location of the caller and receiver could be approximated based on the unique longitude and latitude coordinates for each mobile phone tower. Using the CDRs, a location for each subscriber every time they either made/received a call (or SMS) could be obtained. For each day in the data set, subscribers were assigned a single tower location. If the subscriber made at least one call on that day, then the location of the majority routing tower was assigned. If the subscriber had not made a call on that day, then the location of their most recent routing tower was assigned. This provided a time series of tower location for each subscriber on each day. As done in previous studies, trips are calculated by observing when a subscriber’s tower location has changed from the previous day [7]. However, to compare the mobile phone data to the census data, we aggregated towers to the county-level based on the tower’s location. Thus, only trips between towers in different counties were considered. For each trip measured, the duration of the trip was calculated by counting successive days in the new location.

### Analyses

#### Comparisons between mobile phone and census data

The number of trips derived from the mobile phone data that fell within various trip duration brackets (see legend for Table 1) were calculated. For the various trip durations, the absolute number of trips between all pairs of counties from the mobile phone data were calculated and compared to the census data using linear regression and Pearson’s correlation coefficient. The percentage of county *m*’s population who has traveled to county *n* was calculated. From these values, we ranked each flower based on this relative movement measure for both the mobile phone and census data. The relative values from both source and destination flows between all pairs of counties were compared to construct a relative ranking. Aside from comparing and quantifying amounts of movement, both absolute and relative, an empirical density distribution was constructed based on the physical distance between counties. Using the centroids of each county, the Euclidean distance between all pairs of counties was calculated and a probability distribution based on trip distance was defined.

**Table 1:** The relationship between mobile phone derived movement variables and national census derived migration variables.

Movement Variable	Adjusted R^2^ (outgoing, relative)		Adjusted R^2^ (incoming, relative)			Percentage of Total Movements	
**Len. Week**	0.5634		0.4575			87%	
**Len. Bi-Week**	0.5785		0.4558			6%	
**Len. Month**	0.6063		0.4585			3.9%	
**Len. 2 Months**	0.6413		0.485			2%	
**Len. 3 Months**	0.6555		0.4834			0.5%	
**Len. 4 Months**	0.6652		0.4477			0.2%	
**Avg. Daily**	0.4461		0.3244				
**Avg. Weekly**	0.5962		0.4601				
**Avg. Bi-Weekly**	0.5964		0.453				
**Avg. Monthly**	0.6036		0.4504				
**Yearly**	0.4461		0.3234				
**Len. Bi-Week**		0.5785		0.4558	6%		
**Len. Month**		0.6063		0.4585	3.9%		
**Len. 2 Months**		0.6413		0.485	2%		
**Len. 3 Months**		0.6555		0.4834	0.5%		
**Len. 4 Months**		0.6652		0.4477	0.2%		
**Avg. Daily**		0.4461		0.3244			
**Avg. Weekly**		0.5962		0.4601			
**Avg. Bi-Weekly**		0.5964		0.453			
**Avg. Monthly**		0.6036		0.4504			
**Yearly**		0.4461		0.3234			

The total outgoing and incoming flows from movement between counties were quantified. Movement variables were defined for both various trip durations and the average number of trips over different time frames. All trip duration variables (Len. Week – Len. 4 months) measured the total number of trips that lasted up to the variable name, i.e. Len. Week measures trips lasting up to one week. The average number of trip variables (Avg. Daily – Yearly) measures the trips for various time frames, i.e. Avg. Daily measures the average number of trips each day. For each movement variable, these values were ranked and compared with the ranked values from the total outgoing/incoming movement of individuals from the national census. The census measured responses to the question, ‘where did you live one year ago?’. A linear regression was used to quantify the relationship with adjusted R-squared values presented. Note for all movement variables, p<0.0001.

#### Gravity-type spatial interaction model

The gravity model is one of the most well studied spatial interaction models, where the modeled number of trips between locations *x* and *y*, *N_x,y_* is described by
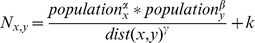
where *population_x_, population_y_* are the populations of locations *x* and *y* and *dist(x,y)* is a function of the distance between *x* and *y*. The exponents, 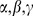
and intercept *k* were obtained from fitting the model to actual data using a generalized linear model with a Poisson specification [28]–[29]. It assumes that the only factors to estimate movement are locations, measured by the physical distance between locations, and importance, measured by the population size at each location. The simplicity of this model makes it a commonly used method to approximate movement between locations using empirical data. The exponents for separate gravity models describing each type of movement using population estimates from the census and Euclidean distance between the centroids of each county were estimated.

## Results

### Comparison of Inter-county Movements between Mobile Phone and Census Data

Using mobile phone call data records (CDRs) from Kenya in 2008–2009, we quantified the average inter-county movement of each individual over a variety of time scales, as well as the number of trips lasting various durations of time (see Methods). We compared these movements with the inter-county movements measured by Kenyan census data. We first analyzed absolute levels of movement between counties. For every time period, mobile phone data greatly overestimated census movement on average between one and four orders of magnitude (Figure S1A, Table S1). Using linear regression, we found that the closest match to the census data were trips lasting longer than three months, but less than four months (adjusted R^2^ = 0.404, p<0.0001) (Figure S1B). Total movement better fit the census data with adjusted R^2^ values ranging from 0.134 to 0.404, all with significant p-values. The poor correlations between absolute numbers of movements derived from census migration data and mobile phone usage data are unsurprising given the different types of movements over differing timescales that each is capturing.

**Figure 1:**
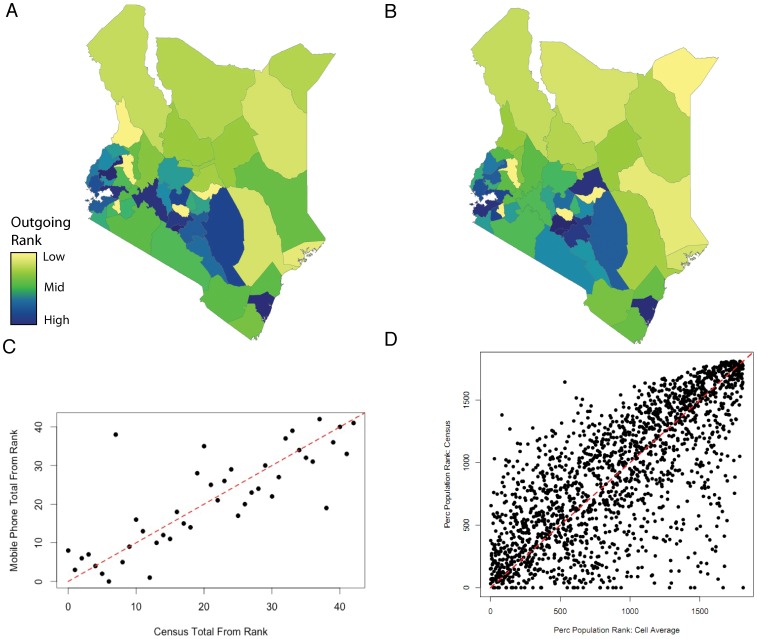
A comparison of the ranked estimates of movement. Counties in Kenya are colored according to the total outgoing rank from A) mobile phone derived movement data (the number of trips between 2 and 3 months, for example movements relevant for studying infectious diseases where transmission varies seasonally, such as influenza) and B) census derived migration data. The actual values are shown in C) with the one-to-one x-y line shown in red. D) The percentage of the population moving between all pairs of counties. For each movement variable, absolute outgoing movements were weighted by the percentage of the population moving to each destination. For both census migration data and mobile phone movement data (the number of trips between 2–3 months), a ranked value was calculated (adjusted R-squared = 0.5421, p<0.001).

However, the relative ordering of counties by movement from the two datasets was strikingly similar. Counties were ranked based on the sum of outgoing and incoming trips for both the mobile phone and census data. The linear fits were strong and significant (adjusted R^2^ values fell between 0.45–0.67 and 0.32–0.60, all with significant p-values for total trips and average number respectively) (see Table 1). Figure 1 shows county level maps colored by their ranked outgoing sum number of trips from A) mobile phone usage data and B) census migration data, displaying the clear correlation between the two ranked values. Figure 1C shows the fit, also for the sum of outgoing trips. Both mobile phone data and census data rank counties were similarly based on total movement incoming/outgoing from each county. Moreover, both the total incoming and outgoing trips were correlated with the county population (Pearson’s correlation coefficient for total incoming = 0.657, for total outgoing = 0.664, p-value<0.001 for both cases). A flow rank computed from mobile phone data compared to a census derived flow ranking was also considered. The relative percentage of county *m*’s population travel to county *n* was calculated. From these values, we ranked each flow based on this relative movement measure for both the mobile phone and census data. The flow ranks were a much closer fit than the absolute movement values (adjusted R^2^ = 0.542, p<0.0001) (Figure 1D). The ranked values of movement involving rural counties were strongly correlated (urban to rural: correlation coefficient = 0.578, p<0.0001, rural to rural: correlation coefficient = 0.53, p<0.0001, rural to urban: correlation coefficient = 0.365, p<0.0001). However, ranked movements between urban counties were not significantly correlated (correlation coefficient = 0.447, p = 0.109) (see Figure S2, Tables S2 and S3).

### Distance Comparison

**Figure 2:**
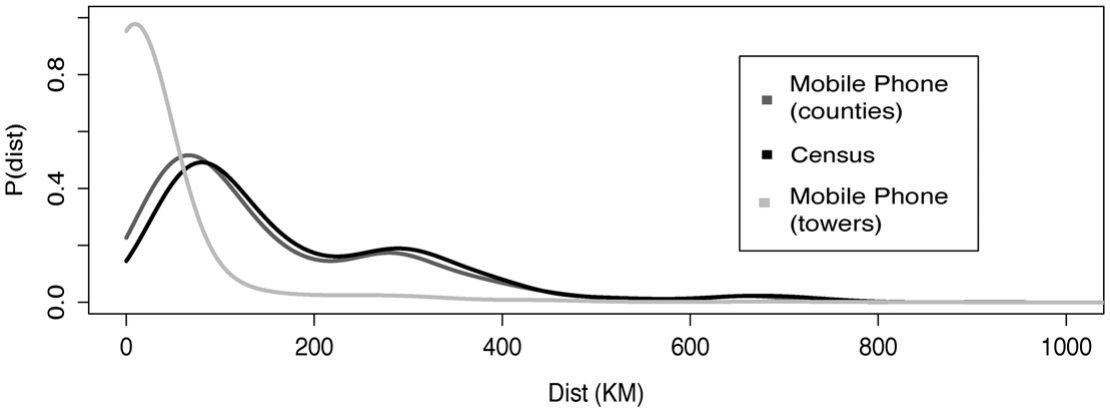
The distribution of trip durations between counties from mobile phone derived movements and census derived migrations. The probability of a trip of various distances for both the census-derived migration data and mobile phone usage data (number trips lasting between 2 and 3 months) was calculated.

From the mobile phone data, relatively small spatial scale movements between mobile phone towers can be quantified. The average journey distance on the mobile phone tower level was 15 km (with a median of 5 km) (Figure 2). When aggregating mobile phone tower movements to the same spatial scale as the national census, the average distance for census movement was higher than the mobile phone data (census mean/median: 182/127 km, mobile phone mean/median: 160/106 km), due to the size and shape of counties and the use of centroids to represent them (see Methods). However, both distributions for the frequency of trips for various distances from both sources of data were similar (Kolmogorov–Smirnov statistic: 0.1168, p<0.0001) [30]. Thus, although census and mobile phone derived movement estimates are not comparable at the absolute level, the likelihood of trips for various distances are similar. In particular, the census data is able to approximate well the likelihood of shorter distance trips than the absolute number of trips quantified using the census data would suggest. The utility of census-derived migration data can therefore be extended to estimate reliably the probability of residents making trips at a range of distances for frequencies substantially shorter than the timescales of a year as used in many census questionnaires.

### Gravity Model Parameterization from Census Data

**Figure 3:**
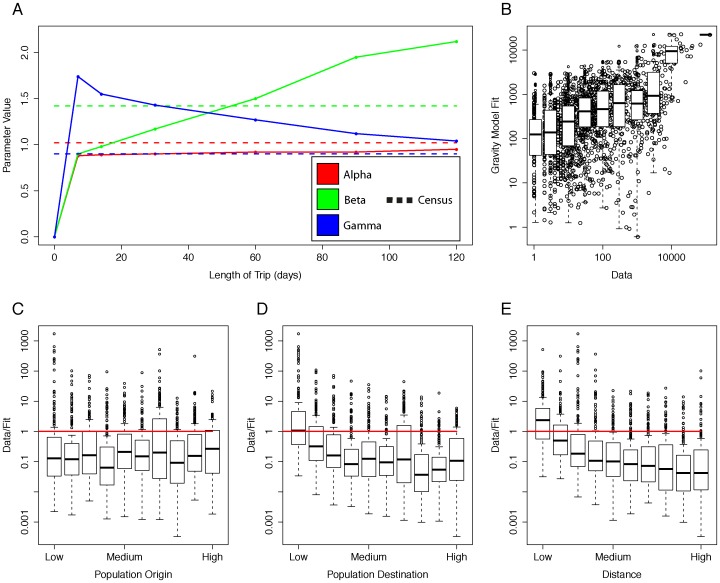
Gravity-type spatial interaction model fits for the mobile phone usage data. Gravity models were calibrated for each movement variable. A) The parameter values for 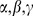 are shown from the fit for various trip durations. Each parameter value from the census data is shown in the corresponding color as a dotted line. A gravity model was calibrated to fit the number of trips between counties lasting between 2 and 3 months. B) The actual data versus the gravity model fit is shown in the figure (Data/Fit). The ratio of true data to the results of the fitted model are shown broken down by C) population at the origin county, D) population at the destination and E) the distance (in kilometers) between the origin and destination. The model underestimates movements from low population counties (both as an origin and destination) and shorter trips.

Finally, the potential for using census-derived migration data as a basis for modeling population movements at differing temporal scales using a gravity-type spatial interaction model was examined. Table S4 presents the estimated exponents, along with the corresponding reduction in deviance fit for each type of movement, including those from the census data. The census data derived migrations showed the smallest reduction in deviance (55.12%), whereas average daily and yearly movement derived from the phone usage data both reduced deviance by 80%. Unsurprisingly, as the duration of journey increases (from one week to 3–4 months), the exponent on the destination’s population, 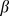, increases, whereas the distance exponent, 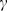, decreases (see Figure 3A). This implies that as the duration of a journey increases, the destination becomes more important in determining the number of trips, while the distance to the destination becomes less important. Figures 3 B–E and S3 A–D show the fit of gravity models from the mobile phone data and census data. In general, the resulting fit overestimates the actual travel. For low population counties and trips over a shorter distance, the gravity model underestimates this travel, however.

## Discussion

Novel approaches to quantifying human movement patterns across spatial and temporal scales continue to increase our understanding of the magnitudes, directions, and drivers of travel. Mobile phone usage data [7], [21]–[27], GPS tracking [2], [31], and satellite imagery [32] are enabling advancements in our understanding of movement dynamics, especially in low-income settings. Such data and analyses are limited to specific locations or countries and timescales, however, and often involve confidential data that cannot be widely shared. If movement patterns in low-income settings are to be quantified and better understood across large areas and a variety of timescales, there is a need to make better use of existing widely and regularly collected data, such as migration data from national censuses. However, the ability of such data sets to generalize to other scales of movement across temporal scales has not been previously analyzed. Here we have shown that census migration data can be used as a surrogate for features of short-temporal scale, more frequent movements.

In absolute terms, unsurprisingly, there are poor correlations between census derived migration data and phone derived movement data due to the differing aspects of movement measured. Migration data obtained for national population and housing censuses are focused on describing permanent changes in residence, whereas phone usage data capture all types of movements, from those permanent residential changes, to seasonal movements, occasional long distance travel and regular routine movements [2], [33]–[34]. However, it is clear that in terms of the relative strength of connections across all temporal scales of movement, strong correlations exist. The reasons behind this are likely many and varied, including social motivations, e.g. areas of economic opportunities and family ties, or physical features, e.g. transportation accessibility and hindrances by natural barriers such as mountains or lakes [19], [35]. The strong relative relationship between the shorter temporal scale movement patterns and the census-derived migrations remains across all temporal scales investigated. This offers practitioners the possibility of extending the utility of census data to obtain relative estimates of movement on smaller time scales for multiple applications. These may include the mapping of clusters of regions that are relatively strongly connected by movements at relevant timescales for disease control and elimination planning purposes [7], [21], [33], the identification of relatively poorly connected/isolated regions [33], and economic development planning for infrastructure improvement [36]. Moreover, the gravity models exponents (Table S4) and, in particular, the relationship between the exponents from various types of phone-derived movement and exponents from the census migration data, can be used to approximate a variety of types of movement when only a national census is provided (see Figure 3A). Generally, the ratios between these temporally varying types of movement and the census migration data may enable more detailed movement estimates to be obtained after refitting a gravity model to the location of interest.

It is clear that while the findings here illustrate the potential for census migration data to be used to represent shorter time period movements, there exist uncertainties and caveats that must be acknowledged before this is undertaken. While the gravity-type spatial interaction model fits result in a large deviance reduction, there still exists much variation unaccounted for, some of which can be explained through the addition of extra demographic, socioeconomic and environmental variables [19]. The model performs poorly for travel among less populated counties and for trips over short distances. The exact nature of this variation remains to be fully explained, however, and extrapolation to other countries that have different drivers of movement will be inherently uncertain. Both sources of data used in this study have inherent limitations, which are well documented elsewhere [37]–[38]. Arguably, mobile phone data provides some of the most detailed human movement data available on a national scale. Nonetheless, such data have inherent biases and are not necessarily representative of the population [37]. In addition, census migration data has uncertainties that can arise from the interpretation of migration questions by respondents, actual time within the year of moving, and inability to fully capture mobile communities of individuals such as migrant workers or nomadic peoples. Finally, the analyses here were limited by the spatial scale of the national census and, thus, do not leverage the refined movement patterns available from the mobile phone data or address any heterogeneity in movement patterns within a county.

Increasing interest in the spatial modeling of infectious diseases [4]–[5], [8], [38], geographical drivers of economic development [36] and access to basic services [9], [39]–[41] are driving a rising demand for empirical data and models of human movement patterns across multiple spatial and temporal scales. This demand is in turn accelerating the exploitation of traditional data sources, such as census, commuting and household survey data, as well as the development of novel approaches based on data sources not previously available, including mobile phones and GPS tracking devices [2]. Each of these data sources has inherent strengths and weaknesses, ranging from variations in sample sizes, spatiotemporal coverage and resolution, and ease of data collection and availability. Great potential exists to combine these differing datasets in a range of ways to build on the strengths of each and produce a more complete understanding of human movement patterns across spatial and temporal scales, as demonstrated in these analyses. Such approaches represent the aims of a wider initiative, The Human Mobility Mapping Project (www.thummp.org), focused on improved quantification of human movement patterns in low-income regions and the development of open access models to describe them.

## Supporting Information

Figure S1 **Comparisons between mobile phone data derived movements and census migrations.** A) For each pair of counties, the average number of trips lasting between 2 and 3 months was calculated from the mobile phone data. This number is compared with the amount of movement from the national census data. The x–y line is shown in red, indicating the overestimation by mobile phone data. B) The relationship between each absolute values of movement from each movement variable was compared to the census data. Adjusted R^2^ values were produced using a linear regression.
(PDF)

Figure S2 **The relationship between mobile phone movement patterns and the census data for counties partitioned by urban, rural movements.** Counties were classified as either urban or rural and all movement patterns are segmented based on the origin and destination classification. Mobile phone data (here, trips lasting between two and three months) overestimated the census data with the dotted lines showing the x–y line.
(PDF)

Figure S3 **The resulting fit from the gravity model describing the census data.** A) The actual data versus the gravity model fit. The ratio of true data to the results of the fitted model are shown broken by A) population of the origin B) population of the destination and C) the distance (in kilometers) between the origin and destination.The gravity model under estimates movements from low population counties (both as an origin and destination) and shorter trips. In general, the model overestimates the amount of travel.
(PDF)

Table S1 **The ratio between mobile phone data and census data for all movement variables.** For all movement variables quantified using the mobile phone data, we compared the ratio of this data to the census data. Minimum and maximum values form the 90% quantile interval. For all types of movement, the mobile phone overestimates the census data.
(DOCX)

Table S2 **The ratio of mobile phone movement values and the census movement for trips divided by county type.** Movements are partitioned according to trips A) from urban counties to rural counties B) between urban counties C) from rural counties to urban counties and D) between rural counties. Minimum and maximum values form the 90% quantile interval. For all movement variables except some instances of trips lasting between three and four months and the average number of daily trips, mobile phone data overestimates the census data. For trips between urban counties, the mobile phone data has the largest overestimation of the census data.
(DOCX)

Table S3 **The correlation between mobile phone movement patterns and the census data for counties partitioned by urban, rural movements.** A Pearson’s correlation coefficient was used to quantify the relationship between mobile phone movements and the census data. Significant correlation coefficients (p<0.05) are marked with an asterisk. Movements were partitioned by the urban/rural category of the origin and destination. In general, the relationship between both sources of data is the strongest between urban to rural trips and rural to urban trips. The relationship of movement between urban counties is only significant for trips lasting between two and four months.
(DOCX)

Table S4 **Coefficients and fit for gravity models.** For each movement variable, a gravity model was fit using populations for the origin and destination as well as the Euclidean distance between the origin and destination.
(DOCX)

Text S1 **Supplementary information text.**
(DOCX)
