# Supplementary figures and images for: The Use of Census Migration Data to Approximate Human Movement Patterns across Temporal Scales
Source: PLoS One. 2013 Jan 9;8(1):e52971. doi: 10.1371/journal.pone.0052971 (PMC3541275; doi:10.1371/journal.pone.0052971)

A

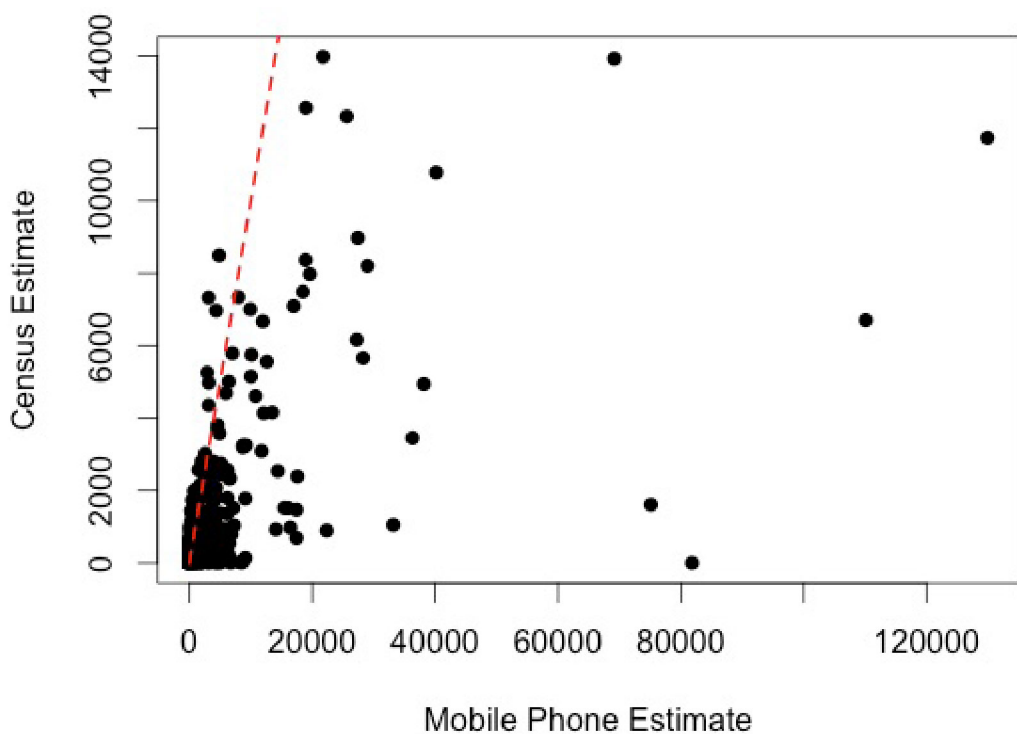

B

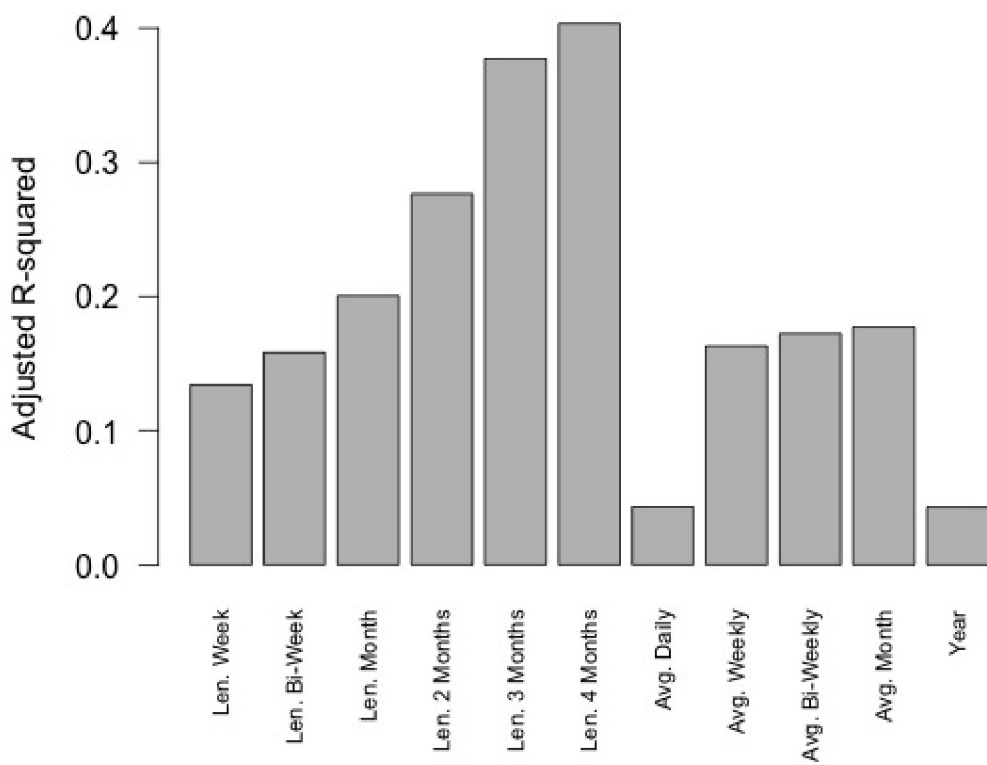

Supplement: Figure S1 — Comparisons between mobile phone data derived movements and census migrations. A) For each pair of counties, the average number of trips lasting between 2 and 3 months was calculated from the mobile phone data. This number is compared with the amount of movement from the national census data. The x–y line is shown in red, indicating the overestimation by mobile phone data. B) The relationship between each absolute values of movement from each movement variable was compared to the census data. Adjusted R2 values were produced using a linear regression. (PDF) [file pone.0052971.s001.pdf]

**Rural->Rural**

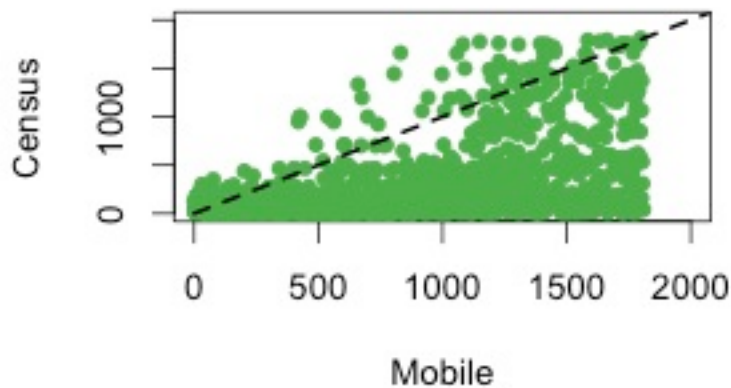

**Rural->Urban**

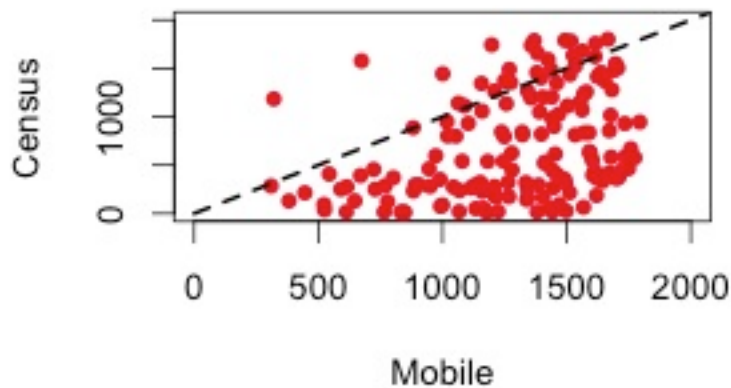

**Urban->Urban**

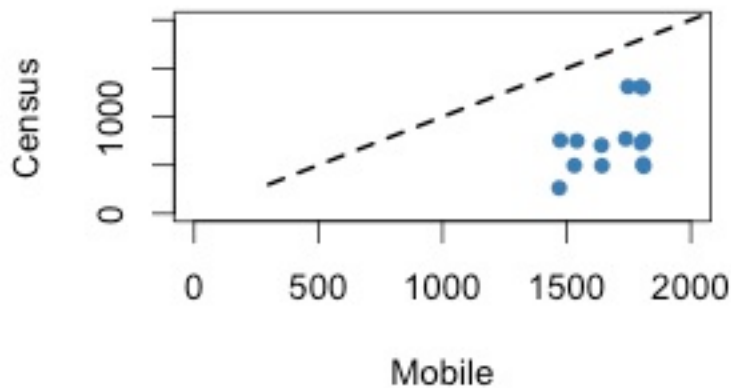

**Urban->Rural**

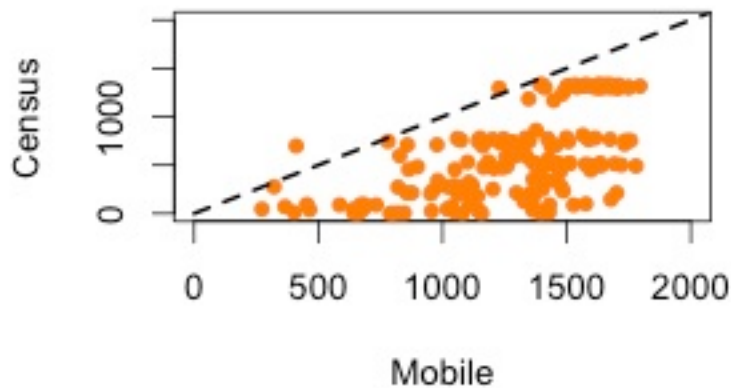

Supplement: Figure S2 — The relationship between mobile phone movement patterns and the census data for counties partitioned by urban, rural movements. Counties were classified as either urban or rural and all movement patterns are segmented based on the origin and destination classification. Mobile phone data (here, trips lasting between two and three months) overestimated the census data with the dotted lines showing the x–y line. (PDF) [file pone.0052971.s002.pdf]

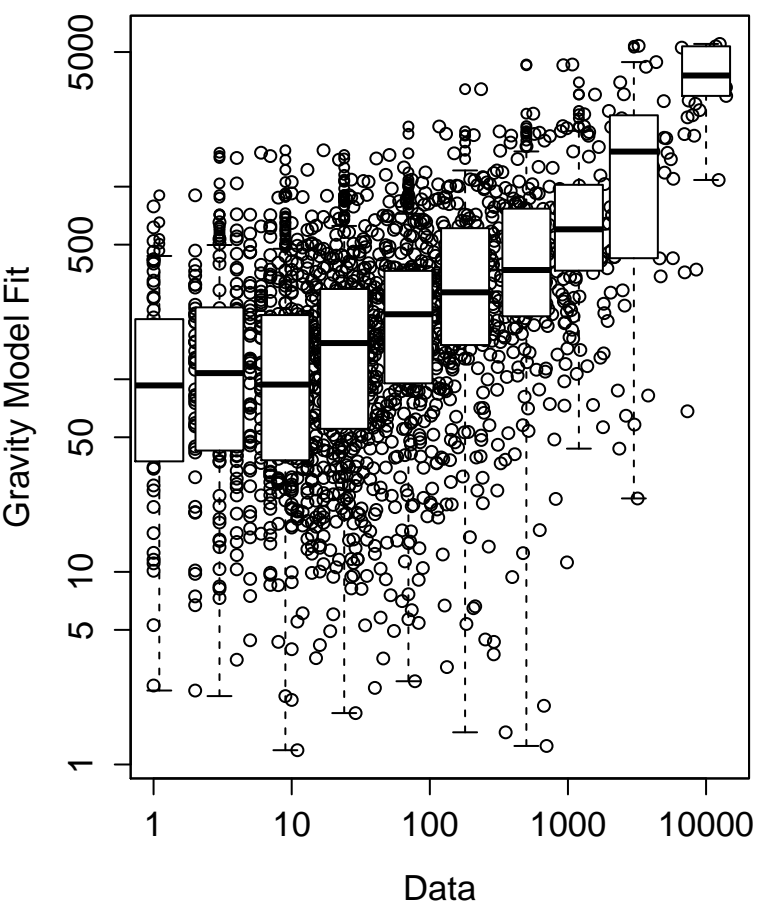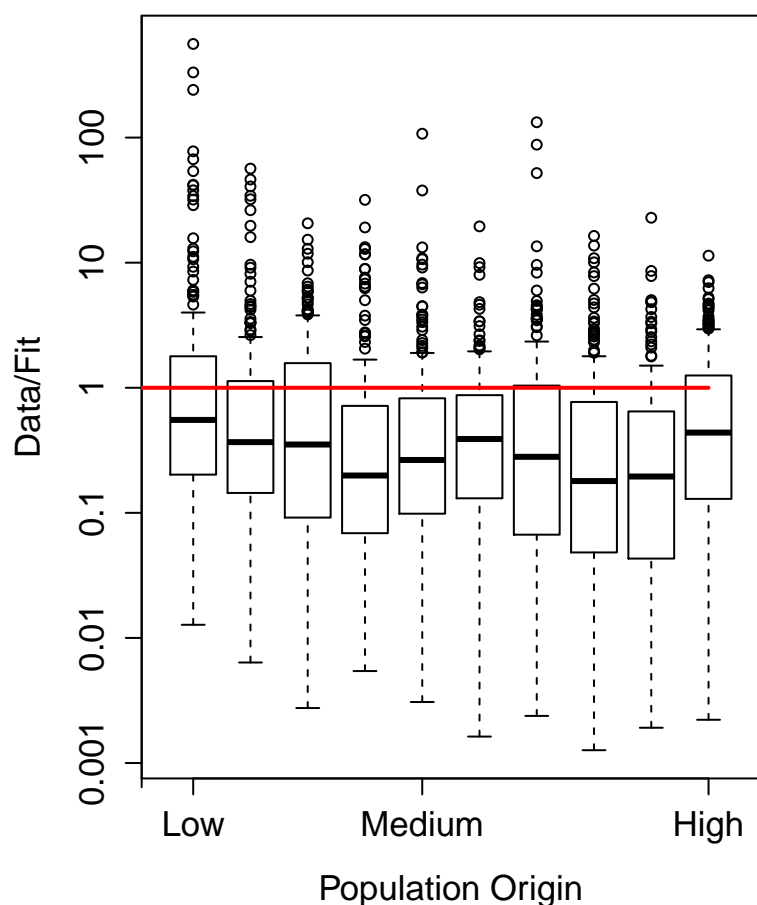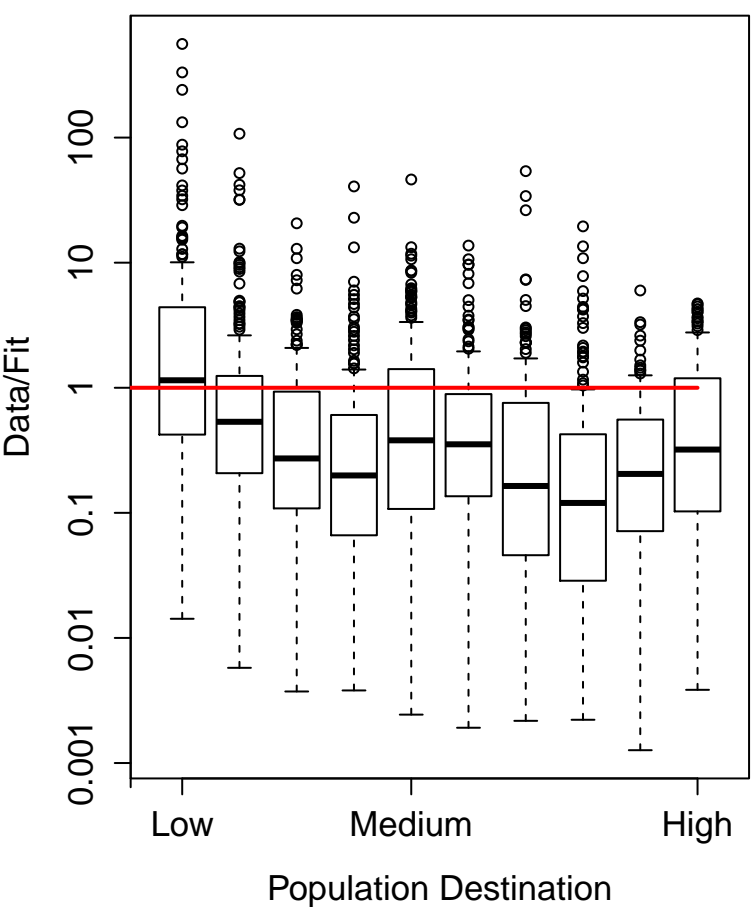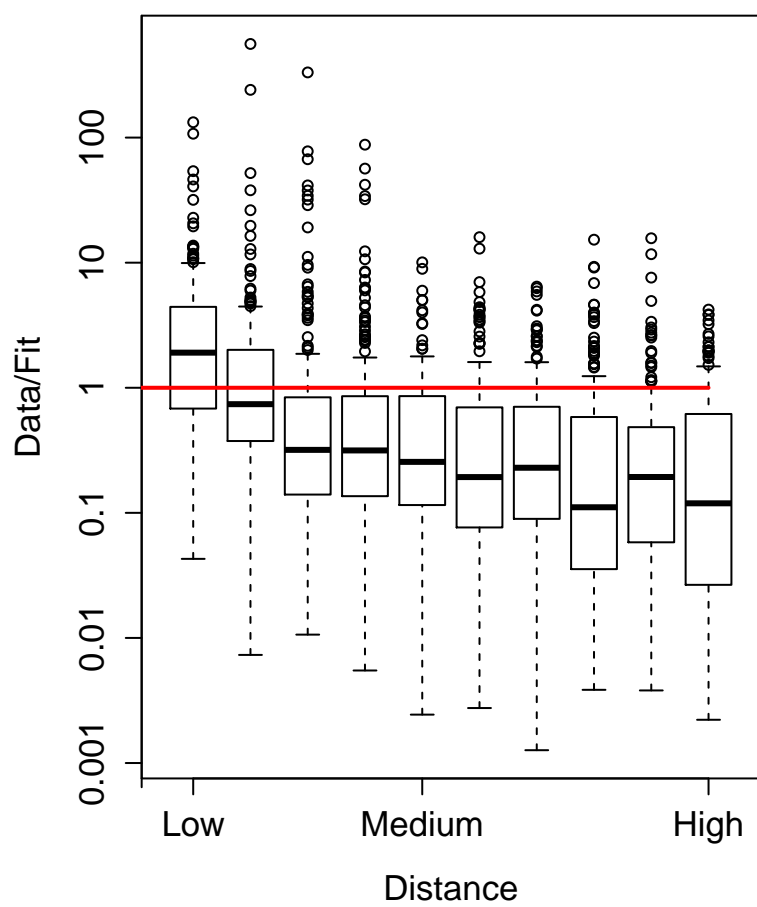

Supplement: Figure S3 — The resulting fit from the gravity model describing the census data. A) The actual data versus the gravity model fit. The ratio of true data to the results of the fitted model are shown broken by A) population of the origin B) population of the destination and C) the distance (in kilometers) between the origin and destination.The gravity model under estimates movements from low population counties (both as an origin and destination) and shorter trips. In general, the model overestimates the amount of travel. (PDF) [file pone.0052971.s003.pdf]
