# Supplementary material for: The Use of Census Migration Data to Approximate Human Movement Patterns across Temporal Scales
Source: PLoS One. 2013 Jan 9;8(1):e52971. doi: 10.1371/journal.pone.0052971 (PMC3541275; doi:10.1371/journal.pone.0052971)
Supplement: Table S1 — The ratio between mobile phone data and census data for all movement variables. For all movement variables quantified using the mobile phone data, we compared the ratio of this data to the census data. Minimum and maximum values form the 90% quantile interval. For all types of movement, the mobile phone overestimates the census data. (DOCX) [file pone.0052971.s004.docx]

| **Movement variable** | **Min** | **Median** | **Mean** | **Max** |
| --- | --- | --- | --- | --- |
| **Len. Week** | 1.47 | 41.43 | 1140.37 | 2159.02 |
| **Len. Bi-Week** | 0.29 | 4.24 | 92.69 | 168.50 |
| **Len. Month** | 0.33 | 3.67 | 60.64 | 115.19 |
| **Len. 2 Months** | 0.26 | 2.57 | 30.05 | 59.46 |
| **Len. 3 Months** | 0.04 | 0.69 | 7.81 | 16.05 |
| **Len. 4 Months** | 0.00 | 0.24 | 3.09 | 6.17 |
| **Avg. Daily** | 0.04 | 0.26 | 2.15 | 4.23 |
| **Avg. Weekly** | 2.30 | 13.06 | 101.92 | 262.56 |
| **Avg. Bi-Weekly** | 6.73 | 31.93 | 221.19 | 595.02 |
| **Avg. Monthly** | 9.42 | 43.36 | 281.30 | 750.44 |
| **Yearly** | 2.07 | 50.59 | 704.94 | 1313.87 |
