# Supplementary material for: The Use of Census Migration Data to Approximate Human Movement Patterns across Temporal Scales
Source: PLoS One. 2013 Jan 9;8(1):e52971. doi: 10.1371/journal.pone.0052971 (PMC3541275; doi:10.1371/journal.pone.0052971)
Supplement: Table S2 — The ratio of mobile phone movement values and the census movement for trips divided by county type. Movements are partitioned according to trips A) from urban counties to rural counties B) between urban counties C) from rural counties to urban counties and D) between rural counties. Minimum and maximum values form the 90% quantile interval. For all movement variables except some instances of trips lasting between three and four months and the average number of daily trips, mobile phone data overestimates the census data. For trips between urban counties, the mobile phone data has the largest overestimation of the census data. (DOCX) [file pone.0052971.s005.docx]

**A.**

| **Movement variable** | **Min (0.05)** | **Median** | **Mean** | **Max (0.95)** |
| --- | --- | --- | --- | --- |
| **Len. Week** | 10.60 | 90.80 | 457.00 | 1210.00 |
| **Len. Bi-Week** | 1.36 | 8.86 | 35.10 | 87.60 |
| **Len. Month** | 1.12 | 7.31 | 23.60 | 58.70 |
| **Len. 2 Months** | 0.91 | 4.44 | 12.20 | 29.70 |
| **Len. 3 Months** | 0.24 | 1.24 | 3.23 | 8.05 |
| **Len. 4 Months** | 0.08 | 0.45 | 1.26 | 3.49 |
| **Avg. Daily** | 0.05 | 0.37 | 1.15 | 3.72 |
| **Avg. Weekly** | 6.42 | 21.80 | 57.20 | 145.00 |
| **Avg. Bi-Weekly** | 15.60 | 52.80 | 129.00 | 359.00 |
| **Avg. Monthly** | 21.50 | 71.70 | 169.00 | 475.00 |
| **Yearly** | 13.00 | 129.00 | 400.00 | 1210.00 |

B.

| **Movement variable** | **Min (0.05)** | **Median** | **Mean** | **Max (0.95)** |
| --- | --- | --- | --- | --- |
| **Len. Week** | 68.70 | 314.00 | 1890.00 | 8700.00 |
| **Len. Bi-Week** | 3.75 | 17.80 | 114.00 | 528.00 |
| **Len. Month** | 2.67 | 11.70 | 66.00 | 294.00 |
| **Len. 2 Months** | 1.59 | 6.60 | 29.10 | 121.00 |
| **Len. 3 Months** | 0.41 | 2.11 | 7.25 | 27.00 |
| **Len. 4 Months** | 0.15 | 0.84 | 2.72 | 9.58 |
| **Avg. Daily** | 0.23 | 0.92 | 34.90 | 189.00 |
| **Avg. Weekly** | 9.18 | 28.00 | 115.00 | 505.00 |
| **Avg. Bi-Weekly** | 20.40 | 60.90 | 234.00 | 1010.00 |
| **Avg. Monthly** | 26.40 | 78.00 | 288.00 | 1240.00 |
| **Yearly** | 80.90 | 327.00 | 12600.00 | 67900.00 |

C.

| **Movement variable** | **Min (0.05)** | **Median** | **Mean** | **Max (0.95)** |
| --- | --- | --- | --- | --- |
| **Len. Week** | 4.99 | 34.83 | 125.39 | 429.24 |
| **Len. Bi-Week** | 0.59 | 3.33 | 9.21 | 21.38 |
| **Len. Month** | 0.47 | 3.03 | 6.99 | 17.82 |
| **Len. 2 Months** | 0.27 | 2.16 | 4.42 | 12.93 |
| **Len. 3 Months** | 0.07 | 0.73 | 1.53 | 4.99 |
| **Len. 4 Months** | 0.03 | 0.30 | 0.64 | 2.16 |
| **Avg. Daily** | 0.03 | 0.15 | 0.29 | 1.08 |
| **Avg. Weekly** | 2.70 | 9.72 | 16.14 | 40.12 |
| **Avg. Bi-Weekly** | 7.11 | 22.63 | 36.81 | 84.30 |
| **Avg. Monthly** | 9.42 | 29.64 | 48.04 | 107.49 |
| **Yearly** | 6.57 | 47.60 | 99.66 | 379.30 |

D.

| **Movement variable** | **Min (0.05)** | **Median** | **Mean** | **Max (0.95)** |
| --- | --- | --- | --- | --- |
| **Len. Week** | 1.21 | 35.80 | 1320.00 | 2570.00 |
| **Len. Bi-Week** | 0.24 | 3.80 | 108.00 | 206.00 |
| **Len. Month** | 0.29 | 3.30 | 70.70 | 138.00 |
| **Len. 2 Months** | 0.24 | 2.38 | 34.90 | 67.00 |
| **Len. 3 Months** | 0.01 | 0.60 | 9.03 | 18.00 |
| **Len. 4 Months** | 0.00 | 0.21 | 3.57 | 7.18 |
| **Avg. Daily** | 0.04 | 0.26 | 2.15 | 4.54 |
| **Avg. Weekly** | 2.12 | 12.60 | 116.00 | 310.00 |
| **Avg. Bi-Weekly** | 6.43 | 31.20 | 252.00 | 705.00 |
| **Avg. Monthly** | 8.92 | 42.30 | 320.00 | 960.00 |
| **Yearly** | 1.61 | 44.10 | 691.00 | 1400.00 |
