# Supplementary material for: The Use of Census Migration Data to Approximate Human Movement Patterns across Temporal Scales
Source: PLoS One. 2013 Jan 9;8(1):e52971. doi: 10.1371/journal.pone.0052971 (PMC3541275; doi:10.1371/journal.pone.0052971)
Supplement: Table S3 — The correlation between mobile phone movement patterns and the census data for counties partitioned by urban, rural movements. A Pearson’s correlation coefficient was used to quantify the relationship between mobile phone movements and the census data. Significant correlation coefficients (p<0.05) are marked with an asterisk. Movements were partitioned by the urban/rural category of the origin and destination. In general, the relationship between both sources of data is the strongest between urban to rural trips and rural to urban trips. The relationship of movement between urban counties is only significant for trips lasting between two and four months. (DOCX) [file pone.0052971.s006.docx]

| **Movement variable** | **Urban 🡪 Rural** | **Urban 🡪 Urban** | **Rural 🡪 Urban** | **Rural 🡪 Rural** |
| --- | --- | --- | --- | --- |
| **Len. Week** | 0.64* | 0.31 | 0.57* | 0.38* |
| **Len. Bi-Week** | 0.64* | 0.33 | 0.71* | 0.38* |
| **Len. Month** | 0.66* | 0.38 | 0.77* | 0.39* |
| **Len. 2 Months** | 0.68* | 0.46 | 0.82* | 0.40* |
| **Len. 3 Months** | 0.67* | 0.54* | 0.85* | 0.41* |
| **Len. 4 Months** | 0.65* | 0.56* | 0.85* | 0.41* |
| **Avg. Daily** | 0.65* | -0.01 | 0.66* | 0.34* |
| **Avg. Weekly** | 0.72* | 0.29 | 0.74* | 0.38* |
| **Avg. Bi-Weekly** | 0.73* | 0.29 | 0.75* | 0.39* |
| **Avg. Monthly** | 0.73* | 0.30 | 0.75* | 0.39* |
| **Yearly** | 0.65* | -0.01 | 0.66* | 0.34* |
