# Supplementary material for: The Use of Census Migration Data to Approximate Human Movement Patterns across Temporal Scales
Source: PLoS One. 2013 Jan 9;8(1):e52971. doi: 10.1371/journal.pone.0052971 (PMC3541275; doi:10.1371/journal.pone.0052971)
Supplement: Table S4 — Coefficients and fit for gravity models. For each movement variable, a gravity model was fit using populations for the origin and destination as well as the Euclidean distance between the origin and destination. (DOCX) [file pone.0052971.s007.docx]

| Movement Variable | Intercept  *k* | From Pop $\alpha$  | To Pop $\beta$  | Distance $\gamma$  | Model Deviance | Null Deviance | Deviance Reduction |
| --- | --- | --- | --- | --- | --- | --- | --- |
| Len. Week | -12.21 | 0.88 | 0.90 | 1.74 | 281816500.00 | 846299100.00 | 66.70 |
| Len. Bi-Week | -15.98 | 0.89 | 0.98 | 1.55 | 21932130.00 | 57875650.00 | 62.10 |
| Len. Month | -19.18 | 0.90 | 1.17 | 1.43 | 14597720.00 | 38084850.00 | 61.67 |
| Len. 2 Month | -24.69 | 0.92 | 1.50 | 1.27 | 7630927.00 | 20111880.00 | 62.06 |
| Len. 3 Month | -32.33 | 0.92 | 1.95 | 1.12 | 2399861.00 | 6530470.00 | 63.25 |
| Len. 4 Month | -36.09 | 0.95 | 2.12 | 1.04 | 1023517.00 | 2773135.00 | 63.09 |
| Avg. Day | -16.86 | 0.80 | 0.88 | 2.17 | 545726.50 | 2707119.00 | 79.84 |
| Avg. Week | -18.14 | 1.01 | 1.02 | 1.31 | 20992230.00 | 55481570.00 | 62.16 |
| Avg. Bi-Week | -17.93 | 1.04 | 1.03 | 1.24 | 43792040.00 | 114004500.00 | 61.59 |
| Avg. Month | -18.07 | 1.06 | 1.04 | 1.21 | 54490020.00 | 141482100.00 | 61.49 |
| Avg. Year | -11.08 | 0.80 | 0.89 | 2.19 | 196953400.00 | 969212700.00 | 79.68 |
| Census | -27.05 | 1.02 | 1.42 | 0.90 | 907204.40 | 2021501.00 | 55.12 |
