# Supplementary material for: The Use of Census Migration Data to Approximate Human Movement Patterns across Temporal Scales
Source: PLoS One. 2013 Jan 9;8(1):e52971. doi: 10.1371/journal.pone.0052971 (PMC3541275; doi:10.1371/journal.pone.0052971)
Supplement: Text S1 — Supplementary information text. (DOCX) [file pone.0052971.s008.docx]

**Supplemental Information**

**Ratio of movement values**

Mobile phone data greatly overestimates the movement quantified from the census data with the ratios between the two are shown in Figure S1 and Table S1.

**Urban, Rural movement patterns**

We identified counties as urban or rural based on the percentage of the population considered urban in the county (urbanness values obtained from GRUMP (http://sedac.ciesin.columbia.edu/gpw/)). Counties where at least half of the population was considered urban were designated urban counties and conversely for rural counties. Movement data was partition into trips between urban and urban counties (urban 🡪 urban), urban and rural counties (urban 🡪 rural), rural and urban counties (rural 🡪 urban), and rural and rural counties (rural 🡪 rural). The ratio of movement of mobile phone data to census data is shown in Table S2 and scatterplots are shown in Figure S1. The relationship is quantified using a correlation coefficient shown in Table S3.

**Gravity Model Fits**

The parameters fitted to each type of movement are shown in Table S4.
